# Lateralized response of skull bone marrow via osteopontin signaling in mice after ischemia reperfusion

**DOI:** 10.1186/s12974-023-02980-x

**Published:** 2023-12-09

**Authors:** Chaoran Xu, Qia Zhang, Yi Zhang, Huaijun Chen, Tianchi Tang, Junjie Wang, Siqi Xia, Gao Chen, Jianmin Zhang

**Affiliations:** 1 Department of Neurosurgery, The Second Affiliated Hospital, Zhejiang University School of Medicine, Hangzhou, Zhejiang China; 2 Department of Neurosurgery, The Fourth Affiliated Hospital, International Institutes of Medicine, Zhejiang University School of Medicine, Yiwu, Zhejiang China; 3 Brain Research Institute, Zhejiang University, Hangzhou, Zhejiang China; 4 Key Laboratory of Precise Treatment and Clinical Translational Research of Neurological Diseases, Zhejiang University, Hangzhou, Zhejiang China

**Keywords:** Skull bone marrow, Stroke, Lateralization, Cytokine, Sympathetic nervous system

## Abstract

Skull bone marrow is thought to be an immune tissue closely associated with the central nervous system (CNS). Recent studies have focused on the role of skull bone marrow in central nervous system disorders. In this study, we performed single-cell RNA sequencing on ipsilateral and contralateral skull bone marrow cells after experimental stroke and then performed flow cytometry and analysis of cytokine expression. Skull marrow showed lateralization in response to stroke. Lateralization is demonstrated primarily by the proliferation and differentiation of myeloid and lymphoid lineage cells in the skull bone marrow adjacent to the ischemic region, with an increased proportion of neutrophils compared to monocytes. Analysis of chemokines in the skull revealed marked differences in chemotactic signals between the ipsilateral and contralateral skull, whereas sympathetic signals innervating the skull did not affect cranial bone marrow lateralization. Osteopontin (OPN) is involved in region-specific activation of the skull marrow that promotes inflammation in the meninges, and inhibition of OPN expression improves neurological function.

**Graphical Abstract:**

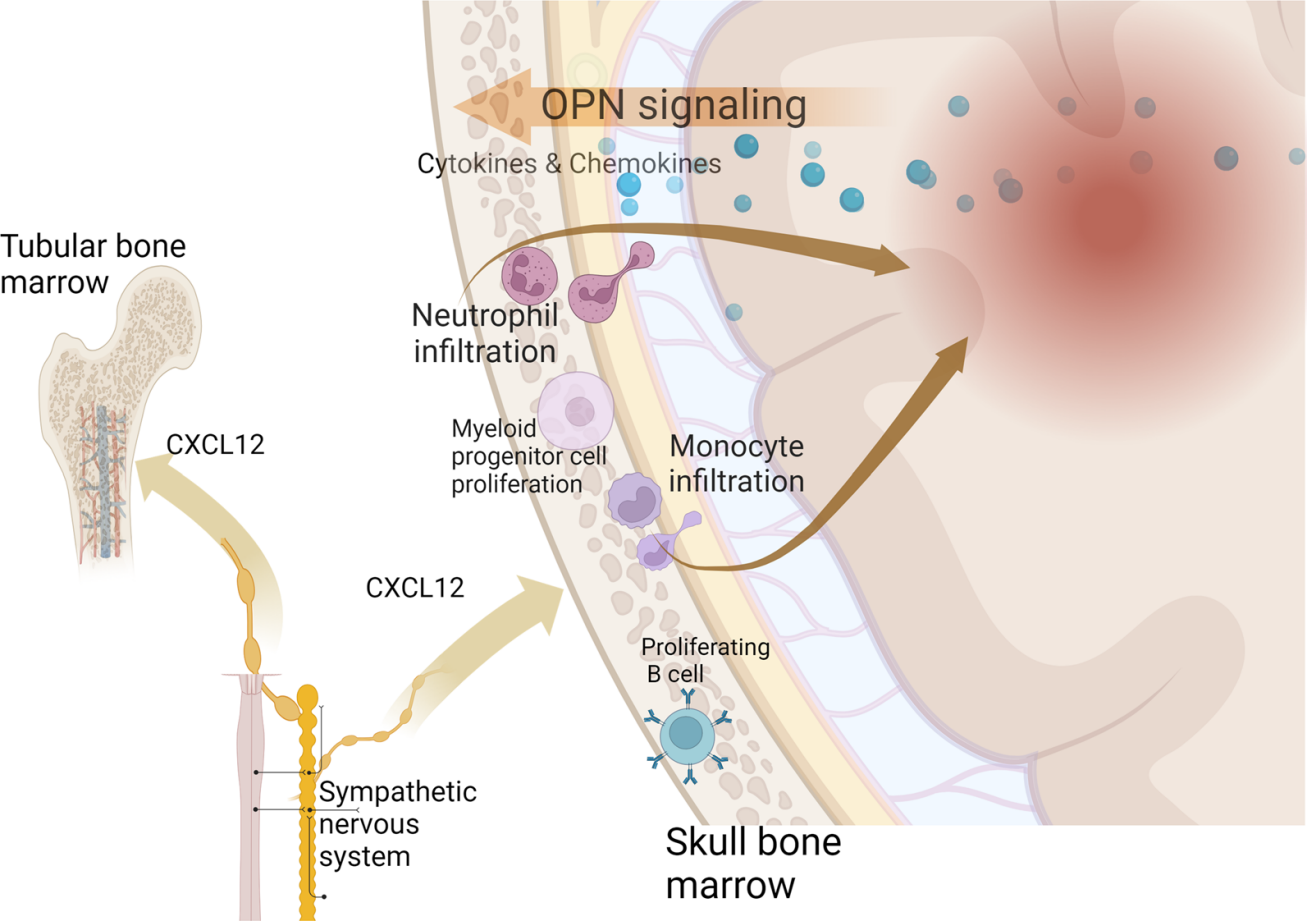

**Supplementary Information:**

The online version contains supplementary material available at 10.1186/s12974-023-02980-x.

## Introduction

The brain is covered by the skull and the meninges [1]. Recent studies in humans and mice suggest that cerebrospinal fluid (CSF) tracers can be enriched in the bone marrow of the skull [2, 3]. Moreover, the skull can be involved in various neurological disorders, providing the central nervous system (CNS) with unique immune cells through direct vascular channels connected to the meninges [3, 4]. Adjacent to the CNS, the cranial marrow, but not the long bone marrow, is the main provider of B lymphocytes to the CNS [5].

Normally, marrow can respond to humoral and neural signals in the setting of CNS disease [6–8]. Adrenergic signals from the sympathetic nervous system modulate the release of immune cells from the bone marrow [9]. In addition to the abovementioned CSF, which may transmit chemotactic signals, the skull has sympathetic innervation that originates from distal branches of some cranial nerves, and neuroimmune cell units may locate the site of injury based on neuroimmune signals [10, 11].

The skulls of humans and mice consist of five principal parts, and each part contains marrow space [12], therefore, each part may respond differently to neuroinflammation. Studies in healthy mice show no difference in fluorescent tracer uptake by dorsal and basal skull regions [4]. However, we are not aware of any previous studies that have explored whether the response of skull bone marrow to CNS injury occurs locally or is widely activated. Here, we used the middle cerebral artery occlusion (MCAO) mouse model to explore whether the cranial marrow response to focal cerebral ischemia is lateral. Our data show that stroke-induced laterality of skull marrow mobilization promotes the differentiation and release of myeloid cells from the ipsilateral bone marrow via osteopontin (OPN/SPP1) signaling.

## Materials and methods

### Animals

All procedures were approved by the Ethics Committee of Zhejiang University and followed the National Institutes of Health guidelines for the Care and Use of Laboratory Animals. Adult male and female C57BL/6J mice (aged 8–10 weeks, weight 21–25 g) were obtained from Charles River Laboratory Animal Co., Ltd. (Zhejiang, China). The mice were kept in a humidity-controlled room (25 ± 1 °C, 12 h light/dark cycle) and were raised with free access to food and water. Detailed animal characteristics are shown in Additional file 1: Table S1. Mice were randomly assigned to experimental groups using random grouping software (Unibiolab, Beijing, China).

### Stroke induction in mice

During the peri-operative period, 1 mg/kg ketoprofen was injected intraperitoneally before surgery. Mice were operated on under isoflurane anesthesia, and body temperature was maintained using a homeothermic monitoring system (RWD Life Science, China). The animals’ pain level during the surgical procedure was monitored by toe clamping. The MCAO sutures were made of silicone-coated nylon thread (MSMC21B080PK50, RWD Life Science, China), which was inserted into the common carotid artery from an incision into the external carotid artery and finally into the middle cerebral artery branch of the internal carotid artery, blocking blood flow without piercing the inner wall of the vessel. The occlusion period was 45 min, after which the MCAO sutures were withdrawn and reperfusion was initiated [13]. Laser Doppler flowmetry (moorVMS-LDF2, Moor Instruments, UK) was used to monitor cerebral blood flow. The interruption of blood flow in the middle cerebral artery was established when cerebral blood flow decreased to less than 25% of the baseline. After 45 min of obstruction, the MCAO sutures were removed, and cerebral blood flow returning to over 50% of the baseline confirmed reperfusion. Mice that did not meet these criteria were excluded from the study. Isoflurane anesthesia was discontinued during reperfusion to observe any neurobehavioral changes. Mice were placed in the heated pad of the homeothermic monitoring system after surgery. For sham-operated group, all procedures were carried out except for the suture which was not inserted into the MCA. Experimenters unaware of group allocation performed MCAO induction in mice.

### Tissue processing

We euthanized the mice using an intraperitoneal injection of an overdose of sodium pentobarbital solution, and once the mice were unresponsive to toe clamping, we proceeded with the subsequent preparation of tissue samples. To study skull bone marrow cells by single-cell RNA sequencing and flow cytometry analysis, the calvarium (frontal and parietal skull) was harvested. After the attached meninges were removed using a microscope, the skull was cut and crushed in HBSS solution, and marrow cells were isolated through a 40-µm Falcon Cell Strainer [4]. The marrow cells from the tibia were collected by a centrifugation-based method [14], which is more efficient for obtaining bone marrow cells than the flushing method [15]. We cut tibiae and placed them in a 0.5 mL microcentrifuge with a hole at the bottom, then we placed the 0.5-mL tube in the 1.5-mL tube and centrifuged bones for 1 min at 2500 × g, RT. Then we add 1 mL RBC lysis buffer (Invitrogen™, USA) to the 1.5-mL tube and resuspend the bone marrow cells. After centrifugation at 340 × *g* for 7 min at 4 °C, samples were resuspended in 300 µL of buffer and kept at 4 °C. The leptomeninges were dissected under the microscope, digested in Liberase/DNAse (Roche Life Science, USA) solution (37 °C, 30 min), and resuspended in Percoll to obtain a single-cell suspension after centrifugation [16].

### Single-cell RNA sequencing

A single-cell suspension of bone marrow cells from a male mouse after MCAO was obtained. After lysing the red blood cells, the suspension was loaded onto the 10 × Genomics Chromium™ system. Reverse transcription and library construction were subsequently performed on the loaded cells. Following the quality inspection of the sequencing libraries, the Illumina HiSeq platform was utilized to sequence the cells with a paired-end 150 sequencing strategy at a depth of 15 million reads per sample.

### Flow cytometry

Different cell types in bone marrow from the skull and tibia were analyzed by flow cytometry on a flow cytometer (Beckman Coulter, Brea, CA, USA) with Cytexpert Software Version 2.4.0.28. The antibodies used in the flow-through assay are listed in Additional file 1: Table S2. The gating strategies are shown in Additional file 1: Fig. S1a.

### Drug administration

SR59230A (MedChemExpress, USA), a β3-adrenergic receptor antagonist, was administered at a dose of 5 mg/kg by intraperitoneal injection 1 h before MCAO. OPN expression inhibitor 1 (MedChemExpress, USA) was administered by intracalvariosseous injection 24 h before MCAO [17]. Fifty micromolars of inhibitor were dissolved in 1% DMSO, and 5 µL of the solution was injected into the skull using a Hamilton syringe [4].

### Western blot analysis

The skull tissue was cut and lysed with RIPA lysis buffer, followed by centrifugation to remove the supernatant (12,000 rpm for 15 min), and then boiled with 5 × loading buffer. Protein samples were loaded onto Bis-gels (GenScript, China) following the manufacturer’s instructions. Finally, digital images were obtained on a chemiluminescence imager (ChemiDoc XRS +, Bio-Rad, USA). The protein expression levels of SDF1/CXCL12 (Cell Signaling, #3740), and OPN/SPP1 (Abcam, ab218237) were quantified using ImageJ.

### Cytokine array

Cytokines in skull marrow were detected using a Proteome Profiler Mouse XL Cytokine Array, which detects over 100 analytes (R&D, ARY028, Minneapolis, MN). The membranes were visualized on X-ray film through chemiluminescence (Super RX-N, Fujifilm, Japan). Analysis of the array membranes was carried out by using ImageJ software to quantify the integrated density.

### Neurological assessment

Modified neurologic severity scores (mNSS) and foot-fault test were used to assess neurological function in mice [18]. The mNSS used a modified version with a total score of 14 [19]. The pre-stroke test of the foot-fault test showed no difference between groups at baseline. Experimenters unaware of group allocation would perform neurobehavioral scoring of mice.

### Statistical analysis

The raw single-cell RNA sequencing data were processed and analyzed using an integrated bioinformatics workflow. Initially, sequence reads in FASTQ files were aligned to the GRCm37 reference genome and gene expression was quantified using the “Cell Ranger” pipeline (version 7.0.1) on Linux. Subsequently, the “Seurat” R package (version 4.3.0) was utilized for downstream analyses. Low-quality cells expressing less than 200 or greater than 99th percentile genes, or more than 25% mitochondrial reads were filtered out. After performing quality control, normalization, and log transformation, the top 2,000 most variable genes were selected as anchors for integration. Principal component analysis (PCA) was then performed to reduce dimensionality, and the first 13 PCs were used to generate t-SNE plots for clustering cells into populations. Marker genes for each cluster were identified using the “FindAllMarkers” function (Wilcoxon rank sum test, fold change ≥ 2 and adjusted *P* ≤ 0.01). Cell populations were annotated using the “scCATCH” R package (version 3.2.1) [20]. B cells, monocytes, and neutrophils were further extracted separately. Within each population, the “FindMarkers” function (Wilcoxon rank sum test, fold change ≥ 1.2 and adjusted *P* ≤ 0.05) was utilized to identify differentially expressed genes between ipsilateral and contralateral cells. Likewise, “Monocle2” (version 2.22.0) was used to infer pseudotime trajectories of single cells. Finally, gene ontology enrichment analysis was performed using the “clusterProfiler” R package (version 4.2.2) with Benjamini‒Hochberg adjustment (adjusted *P* ≤ 0.05), and the results were visualized using “ggplot2” (version 3.4.2). Communication analysis between cells was conducted using CellChat (Version 1.6.1). Normalized expression data from Seurat were input into CellChat. Functions (“computeCommunProb” and “computeCommunProbPathway”) were utilized for interaction calculation with a p value ≤ 0.05 defined as significant interactions. Visualization was accomplished by the “rankNet” and “netAnalysis_signalingRole_heatmap” functions [21]. The results are presented as the mean values ± SDs. The normality of the data was analyzed using the Shapiro–Wilk test. The paired Student’s t test (normal distribution) and paired Wilcoxon tests (nonnormal distribution) were used for comparisons between the contralateral and ipsilateral groups. One-way ANOVA with Tukey’s post hoc test (normal distribution) and Dunn’s multiple comparison test (nonnormal distribution) were used for comparisons among multiple groups. Two-way ANOVA with Bonferroni’s multiple comparison test (normal distribution) was used to analyze the data at different time points. Statistical analyses were performed using Prism 8 (GraphPad, USA).

## Results

### ScRNA-seq analysis of marrow cells from the contralateral and ipsilateral skull after 15 min of stroke reperfusion

**Fig. 1:**
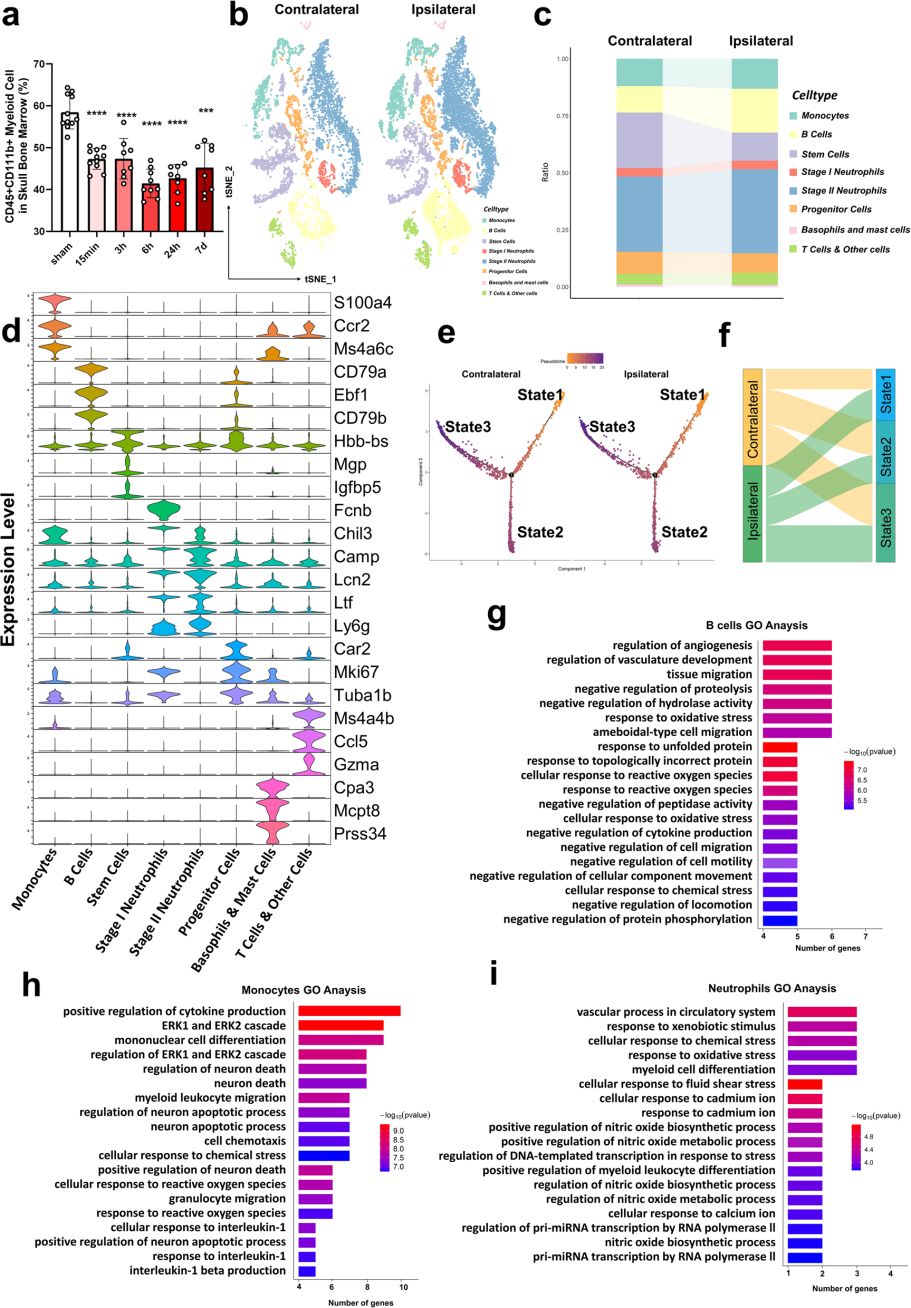
ScRNA-seq analysis of marrow cells from the contralateral and ipsilateral skull after 15 min of stroke reperfusion. **a** Flow cytometry assay of mouse skull marrow myeloid cell proportions 15 min, 3 h, 6 h, 24 h, and 7 d after stroke reperfusion (*n* = 8 to 11 mice per group, one-way ANOVA with Tukey’s multiple comparison). The data are presented as the means ± SDs. ***P < 0.001, ****P < 0.0001, compared to the respective sham group. **b** The tSNE plots show the eight clusters of contralateral and ipsilateral skull marrow cells. **c** Proportion of cell types between the contralateral and ipsilateral groups. **d** Violin plots displaying the distribution and expression of marker genes of the 8 clusters. **e** Differentiation trajectory generated using Monocle 2 with stem cells. Pseudo-time values were calculated and plotted. **f** Sankey diagram showing the ratio of state 1, state 2, and state 3 stem cells between contralateral and ipsilateral sides. **g** Most enriched GO categories of the upregulated (ranked by –log10 (*P* value)) genes in B cells. **h** GO enrichment analysis of upregulated genes in monocytes. **i** GO enrichment analysis of upregulated genes in neutrophils

Previous studies have suggested that bone marrow-derived myeloid cells are the main infiltrating immune cells after cerebral infarction [22]. Therefore, we analyzed changes in the proportion of myeloid cells in the skull during the acute phase of stroke. We observed that the total calvaria CD45^+^CD11b^+^ myeloid cell ratio began to decrease at 15 min after ischemia reperfusion (IR) injury compared to the sham-operated group, and the percentage of myeloid cells was still lower than that of the sham group by day 7 (Fig. 1a). Next, to provide a comprehensive view of the bone marrow changes in the ipsilateral and contralateral skulls, we performed scRNA-seq on sorted marrow cells from skulls at 15 min of reperfusion after MCAO (8307 cells from contralateral and 10,725 cells from ipsilateral). We clustered 8 different cells based on their transcriptomes on UMAP (Fig. 1b). A comparison of the proportions of cell types in the two sides of the skull marrow is shown in Fig. 1c. A total of 8 different cell clusters were identified according to the expression of known marker genes (Fig. 1d), including monocytes (Ccr2, S100a4, Ms4a6c), B cells (CD79a, CD79b, Ebf), stem cells (Hbb-bs, Igfbp5, Mgp), stage 1 neutrophils (Fcnb, Chil3, Camp), stage II neutrophils (Lcn2, Ltf, Ly6g), progenitor cells (Car2, Mki67, Tuba1b), basophils and mast cells (Cpa3, Mcpt8, Prss34), and T cells and other cells (Ms4a4b, Gzma, Ccl5). Stages of the neutrophils were defined by genes upregulated in the most differentiated neutrophil population. Stage 1 includes early neutrophils with high expression of primary granule genes such as Fcnb and Chil3. Stage 2 neutrophils are more mature and begin to express secondary granule genes such as Ltf, Camp, and Ngp [23]. Among the 8 cell clusters defined in our scRNA-seq data, we found a decrease in the proportion of stem cell populations and an increase in the proportion of neutrophils, B cells, etc. (Fig. 1c). We next generated the pseudotime trajectories of contralateral and ipsilateral stem cells, which showed a very similar shape (Fig. 1e). We revealed that the proportion of early-state cells (state 1) increased and exhibited the ability for multiple-lineage differentiation, while cells in state 3 were characterized by a megakaryocyte erythrocyte progenitor (MEP) signature (Fig. 1f, Additional file 2: Table S3). The results of gene ontology analysis of DEGs in contralateral and ipsilateral monocytes, neutrophils, and B cells are presented in Fig. 1g–i. Examples include myeloid cell differentiation in ipsilateral neutrophils, positive regulation of cytokine production and cellular response to chemical response in monocytes, and ameboidal-type cell migration in B cells.

### Comparison of cell numbers and signals between the contralateral and ipsilateral skull

**Fig. 2:**
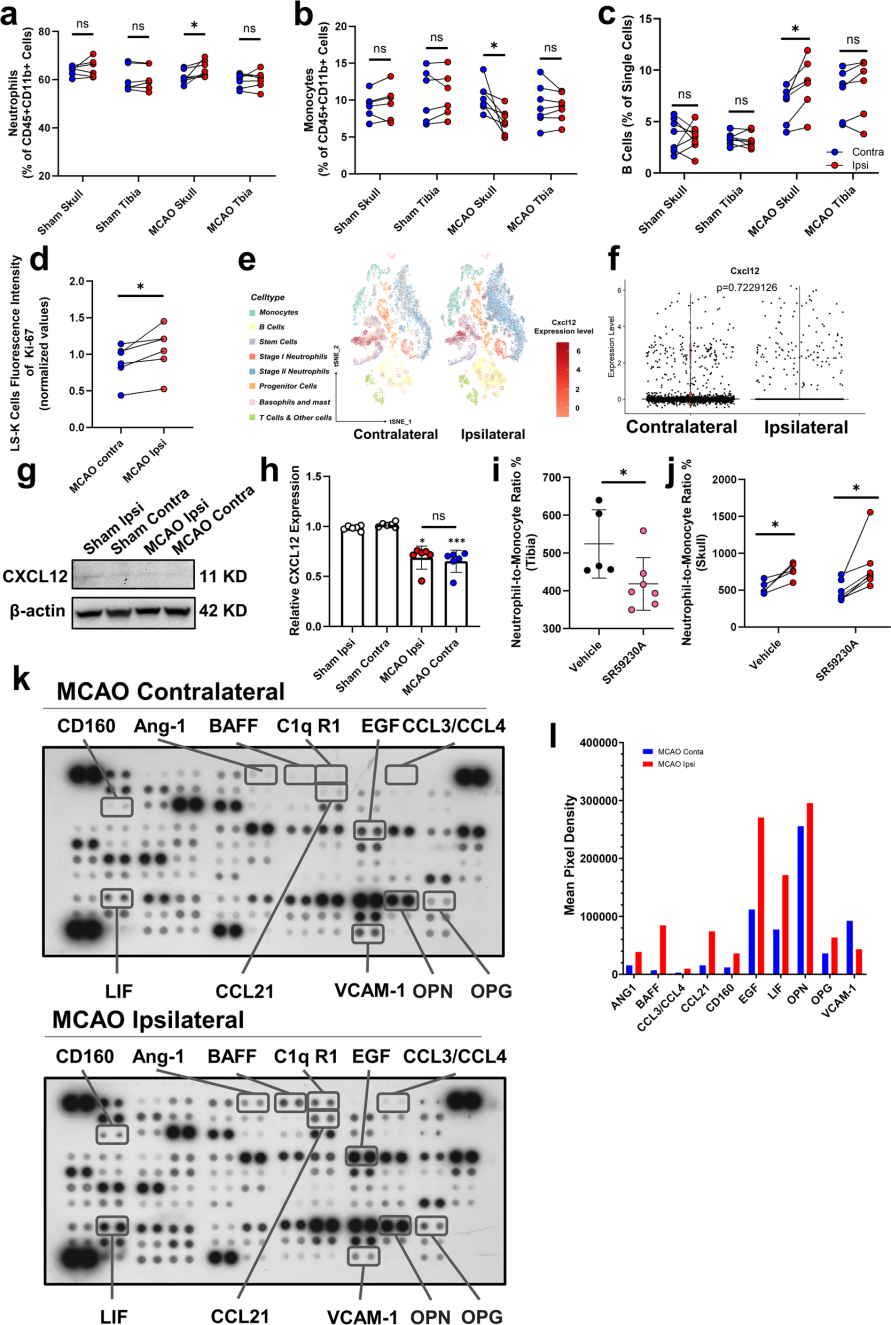
Comparison of cell numbers and signals between contralateral and ipsilateral skulls. **a** Comparison of neutrophil proportions between ipsilateral and contralateral bone marrow in different tissues from male mice. The data are presented as the means ± SDs (paired t test, *P < 0.05, *n* = 6–7). **b** Comparison of monocyte proportions between ipsilateral and contralateral groups in the bone marrow of different tissues from male mice. The data are presented as the means ± SDs (paired t test, *P < 0.05, *n* = 6–7). **c** Comparison of B cell proportions between ipsilateral and contralateral groups in the bone marrow of different tissues from male mice. (paired t test, *P < 0.05, *n* = 6–8). **d** Comparison of Ki67 fluorescence intensity (normalized values) of LS-K cells in the skull marrow. The data are presented as the means ± SDs (paired t test, *P < 0.05, *n* = 6). **e** Single-cell RNA sequencing tSNE plots showing the distribution of CXCL12-positive expression in various cell types of the skull marrow. The distribution of CXCL12 genes is shown in red circles. **f** Violin diagram showing the expression level of CXCL12 between the ipsilateral and contralateral groups (*P* = 0.723). **g** Representative Western blot images of CXCL12 expression in the ipsilateral and contralateral skulls of the sham and stroke groups. **h** Comparison of relative CXCL12 expression levels (normalized to the sham group). The data are presented as the means ± SDs, *P < 0.05; ***P < 0.001. (Dunn’s multiple comparison test, *n* = 6). **i** Neutrophil to monocyte ratio of tibia marrow between the vehicle and SR59230A groups. The data are presented as the means ± SDs, *P < 0.05; (t test, *n* = 5–7). **j** Neutrophil-to-monocyte ratio of ipsilateral and contralateral skull marrow in the vehicle and SR59230A groups. The data are presented as the means ± SDs, *P < 0.05; (paired t test for the vehicle group, *n* = 5; Wilcoxon test for the SR5923A group, *n* = 7). **k** Membranes showing the change in cytokines between the ipsilateral and contralateral skull. **l** The intensities of the spots were quantified. Analysis showed significantly increased cytokines (BAFF, CCL3, CCL21, CD160, EGF, and others) and downregulation of VCAM-1

We next compared the bone marrow of the skull close to the site of injury (MCAO ipsilateral, tibia) with the bone marrow of other sites (MCAO contralateral) to clarify whether changes in the bone marrow occur locally. At 15 min after ischemia reperfusion injury, among those myeloid cells, the proportions of neutrophils and ly6c^Hi^ monocytes in the ipsilateral skull were altered compared to those on the contralateral side (Fig. 2a, b). The proportion of monocytes decreased in the ipsilateral skull marrow, while the proportion of neutrophils increased; furthermore, the neutrophil-to-monocyte ratio exhibited marked laterality (Additional file 1: Fig. S1b). The proportion of B cells in the ipsilateral skull marrow was also increased (Fig. 2c). Similar changes in skull marrow cells were observed in female mice (Additional file 1: Fig. S1c-e). No such laterality was observed in the tibia marrow or skull marrow of the sham group. Furthermore, higher Ki67 signals were also detected on Sca-1-negative progenitor cells (LS-K) in the ipsilateral skull (Fig. 2d). To explore whether neural or chemical signals lead to the immune response in the skull, we first explored the sympathetic regulation of bone marrow. Sympathetic/adrenergic signaling is thought to reduce CXCL12 expression [9], and our previous scRNA-seq data suggested that the CXCL12 gene is predominantly expressed in the cluster of stem cells and did not show laterality (Fig. 2e, f). We then examined CXCL12 protein levels in the skull marrow, and our Western blot studies indicated a decrease in skull CXCL12 after MCAO but no difference between the ipsilateral and contralateral skulls (Fig. 2g, h). To clarify whether sympathetic signals are involved in the lateralization of the bone marrow, using the β3 adrenergic receptor antagonist SR59230A, we found that SR59230A treatment reduced the neutrophil–monocyte ratio in tibia bone marrow (Fig. 2i), but the significant laterality in the neutrophil–monocyte ratio in skull marrow persisted (Fig. 2j). Given that we analyzed the main cytokine and chemokine cell signaling molecules on both sides of the skull, the assay revealed that the levels of multiple cytokines, including BAFF, CCL3, CCL21, CD160, EGF, and others, were highly expressed in the ipsilateral skull (Fig. 2k), and higher secretion of BAFF in the ipsilateral skull was detected (1134.41% increase, Fig. 2l). In addition, a decrease in VCAM-1 expression in the ipsilateral was observed (53.22% decrease, Fig. 2l).

### OPN signaling in the region-specific activation of the skull

**Fig. 3:**
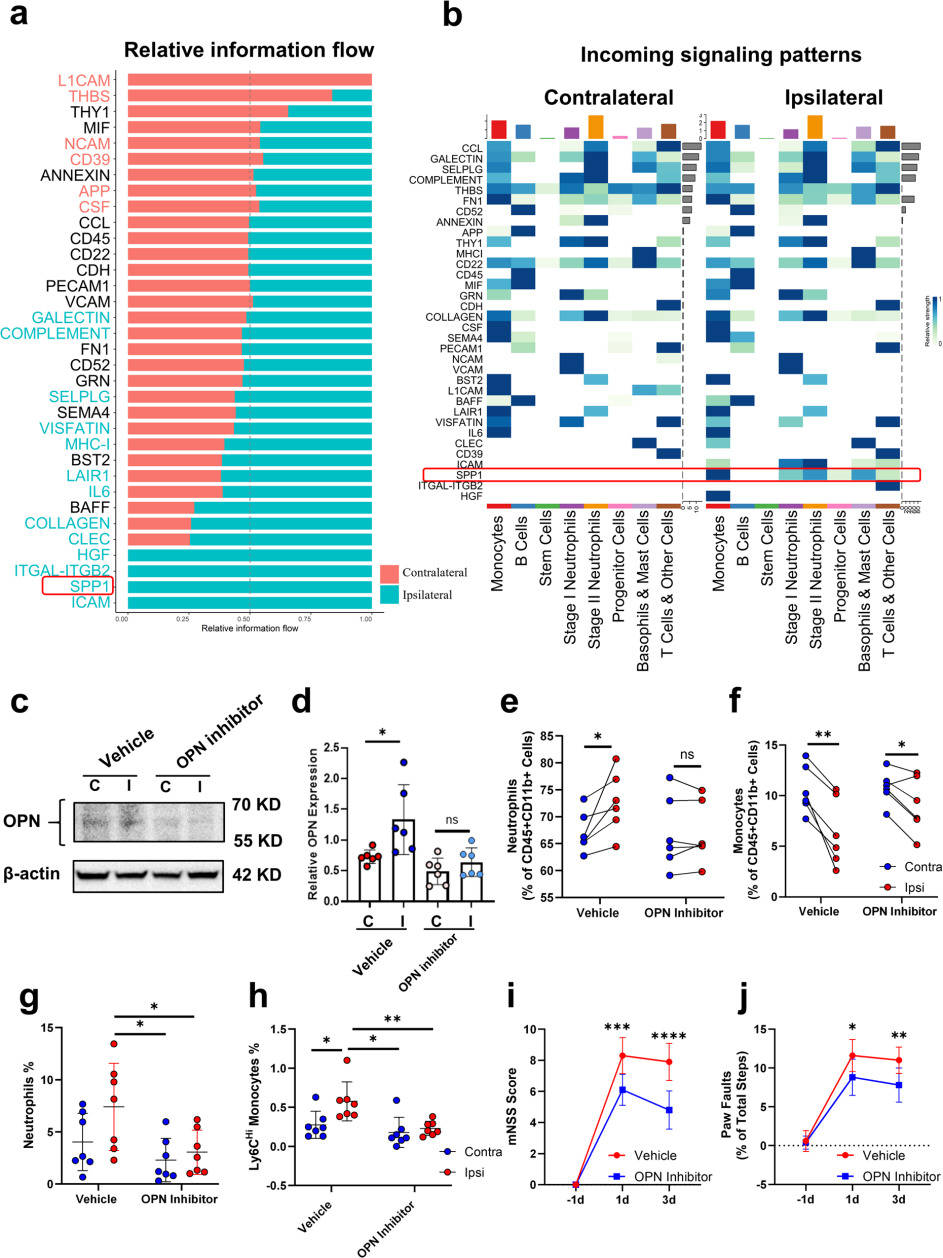
OPN signaling in the region-specific activation of the skull. **a** Signaling pathways of overall information flow in the ipsilateral and contralateral skull. **b** Incoming communication patterns of cells. **c** Representative Western blot images of OPN expression in the contralateral and ipsilateral skulls of the vehicle and OPN inhibitor groups. **d** Comparison of relative OPN expression levels. The data are presented as the means ± SDs, *P < 0.05 (one-way ANOVA with Tukey’s multiple comparison test, *n* = 6). **e** Comparison of neutrophil proportions between different groups of skull marrow. The data are presented as the means ± SDs (paired t test, *P < 0.05, *n* = 6). **f** Comparison of monocyte proportions between different groups of skull marrow. The data are presented as the means ± SDs (paired t test, *P < 0.05, **P < 0.01; *n* = 6). **g** Comparison of leptomeningeal neutrophil proportions between different groups. The data are presented as the means ± SDs, *P < 0.05 (one-way ANOVA with Tukey’s multiple comparison test, *n* = 7). **h** Comparison of leptomeningeal Ly6C^Hi^ monocyte proportions between different groups. The data are presented as the means ± SDs. *P < 0.05, **P < 0.01. (one-way ANOVA with Tukey’s multiple comparison test, *n* = 7). **i** Quantitative analysis of mNSS scores. The data are presented as the means ± SDs. ***P < 0.005, ****P < 0.001 (two-way ANOVA with Bonferroni multiple comparison test, *n* = 10). **j** Quantitative analysis of foot fault percentages. The data are presented as the means ± SDs. *P < 0.05, **P < 0.01 (two-way ANOVA with Bonferroni multiple comparison test, *n* = 10)

We performed CellChat analysis of the contralateral and ipsilateral skull marrow and found that the SPP1(secreted phosphoprotein, OPN) signaling pathway showed increased information flow on the ipsilateral skull, with monocytes, progenitor cells, and neutrophils as the main recipients (Fig. 3a, b). High expression of OPN was also observed in cytokine microarrays (Fig. 2l). OPN expression was increased in the bone marrow of the ipsilateral skull, which was reversed by an osteopontin (OPN) expression inhibitor (Fig. 3c, d). OPN inhibitors primarily reversed the trend of increased neutrophils and monocytes in the affected skull (Fig. 3e, f) and reduced the infiltration of neutrophils and Ly6c^Hi^ monocytes in the meninges after 15 min of ischemia reperfusion (Fig. 3g, h). We then assessed neurological function in the acute phase and found that OPN inhibition via intracalvariosseous injection improved the mNSS score and fore paw function at 1 day and 3 days (Fig. 3i, j).

## Discussion

Previous studies have suggested that skull bone marrow can sense inflammatory signals from the CNS, triggering the production of different leukocyte subtypes [3, 24]. Stronger chemotactic signals were detected in the ipsilateral skull and a greater number of neutrophils, which could be chemotactic to the adjacent site of injury. In line with our observations, in a study of mouse models and stroke patients, neutrophils aggregated in the meninges adjacent to the infarction but were not detected in noninfarcted tissue [25]. Neutrophils that accumulate in the meningeal space and neurovascular unit (NVU) are likely to be subsequently involved in cerebral blood flow and CSF regulation [26]. Moreover, monocytes also exhibit increased proliferative and differentiation activity, and their proportion in the ipsilateral bone marrow is relatively reduced. We speculate that the lateralization of the skull marrow is primarily chemotactic rather than sympathetic nervous system (SNS)-mediated. CXCL12, the downstream effector of the sympathetic–adrenal axis, did not exhibit laterality in the skull marrow. A recent study showed that CXCL12 might regulate white blood cells and their progenitors from the skull after 6 h of IR in a mouse model [4], but did not show laterality in the skull marrow of our study. Ischemic stroke causes strong activation of the SNS and the release of catecholamines, leading to an increase in blood-derived leukocytes [27]. The efferent sympathetic fibers innervating the right and left tibial marrow showed no early differences, whereas the skull marrow exhibited laterality. We speculate that chemokine concentration gradients can affect the skull [28]. Recent studies suggest that the meninges can be divided into four layers, acting as a barrier and regulator of the immune response [29]. Chemokines can cross these meningeal structures to be sensed by the skull and influence the adjacent area of the skull to exhibit lateralization of cell proliferation and differentiation. Our study also suggests that inhibition of OPN signaling reverses skull lateralization. OPN is expressed in myeloid cells and CNS-resident cells and plays an important role in the peripheral innate immune response [30]. Highly expressed levels of OPN in the infarct region were detected in both rats and patients with stroke [31–33]. In addition several studies focusing on OPN seem to show different conclusions, with both OPN recombinant proteins [34] and OPN-neutralizing antibody [31] treatment playing a neuroprotective role. We speculate that OPN may have a detrimental effect in the hyperacute phase of stroke by triggering local immune infiltration, and in the chronic phase of stroke, it may promote functional recovery [35]. The regulatory mechanisms of our skull bone marrow are different from those of other marrow, which emphasizes the importance of local early drug administration. In the bone marrow, immune damage and immunosuppression result from excessive SNS activation after stroke [27, 36], whereas the skull marrow can locally respond to adjacent injury sites rather than undergo extensive activation. In addition, SNS activation could inhibit lymphoid lineage progression (stroke-induced myeloid bias) in the bone marrow; however, after stroke, the skull showed enhanced B cell differentiation, potentially regulating neuroinflammation and promoting functional recovery [37, 38]. We believe that the adjustable regulation of the skull marrow is therefore beneficial and reflects the wisdom of the CNS.

Previous studies on migraine have suggested that the persistent signal from the local cranial marrow may have long-lasting effects on the brain [39], and indeed, we observed that the skull marrow remains unrecovered at 7 d, which may provide a time window for skull marrow intervention and distant brain infarction treatment. Targeting chemotactic signaling has been a trend in stroke intervention [40, 41], as we observed in our experiments that upregulated OPN/SPP1 signaling, while inhibition of OPN reduces neuroinflammation and improves neurological function. The latest bioengineering research presented an intracalvariosseous (ICO) device that can deliver drugs through the skull, providing the possibility of achieving higher local drug concentrations in the target area [42]. As the skull is made up of several bones held together with sutures, there may not be close bony communication with each other [43]. We believe that the blockade of chemotactic signaling targeting a particular bone plate adjacent to the ischemic region can potentially regulate the differentiation and release of specific myeloid/lymphoid cell populations. In humans, hematopoietic bone marrow is found predominantly in flat bones, while in mice, the marrow of long (tubular) bones is also hematopoietic [44, 45]; thus, skull marrow may contribute a greater proportion of infiltrating cells in CNS disease in humans. Therefore, manipulation by modulating OPN signaling in the skull marrow may provide a novel and interesting strategy for treating stroke.

There are several limitations of our study. First, expression of chemokines on microarrays should be validated by other methods. Second, the exact mechanism by which OPN inhibition promotes neurological function in mice needs to be further elucidated. The validity of our findings can be increased by further experiments (e.g., OPN neutralizing antibodies and OPN knockout (KO) mice). Third, the dataset of single-cell RNA sequencing has many cells yet to be further explored.

## Conclusion

The results of the experiments presented herein show that the skull marrow responds rapidly to inflammatory signals of stroke in mouse models. Skull marrow adjacent to the ischemic hemisphere could directly sample signals from the CNS environment; as a result, the ipsilateral bone marrow was highly activated, characterized by proliferating myeloid and lymphoid cell populations. Additionally, marrow cells from the ipsilateral skull show a different response compared to the contralateral skull, which we believe is due to OPN/SPP1 signaling from the ipsilateral hemisphere rather than sympathetic signaling. The OPN signaling increased Ly6c^Hi^ monocytes as well as neutrophil infiltration into the leptomeninges of the ipsilateral. In conclusion, our results reveal previously unrecognized neuroimmune communication of the skull marrow to lateral brain damage.

### Supplementary Information

**Additional file 1. Table S1. **Detailed animal characteristics; **Table S2**. List of antibodies for flow cytometry and Immunofluorescence; **Fig. S1. a** Gating strategy for the flow cytometry assay. **b** Neutrophil-to-monocyte ratio between the ipsilateral and contralateral groups in the bone marrow of male mice. The data are presented as the means ± SD (paired t test, *P < 0.05, n=6-7). **c** Comparison of neutrophils between ipsilateral and contralateral groups in the bone marrow of different tissues of female mice. The data are presented as the means ± SD (paired t test, **P < 0.01, n=6). **d** Comparison of monocytes between ipsilateral and contralateral groups in the bone marrow of different tissues of female mice. The data are presented as the means ± SD (paired t test, **P < 0.01, n=6). **e** Neutrophil-to-monocyte ratio between ipsilateral and contralateral skull marrow of female mice. The data are presented as the means ± SD. (paired t test, **P < 0.01, n=6); Blot images and X-ray image of array.

**Additional file 2. Table S3. **Gene ontology (GO) analysis on three states of stem cell populations.

## Data Availability

All data generated or analyzed during this study are included in this article (and its additional files) or are available on request from the corresponding author.

## Abbreviations

CNS: Central nervous system
CSF: Cerebrospinal fluid
ICO: Intracalvariosseous
IR: Ischemia reperfusion
MCAO: Middle cerebral artery occlusion
MEP: Megakaryocyte erythrocyte progenitor
mNSS: Modified neurologic severity scores
OPN: Osteopontin
SNS: Sympathetic nervous system
SPP1: Secreted phosphoprotein 1
