# Supplementary material for: Lateralized response of skull bone marrow via osteopontin signaling in mice after ischemia reperfusion
Source: J Neuroinflammation. 2023 Dec 9;20:294. doi: 10.1186/s12974-023-02980-x (PMC10710724; doi:10.1186/s12974-023-02980-x)
Supplement: Supplementary file 1 — Additional file 1. Table S1. Detailed animal characteristics; Table S2. List of antibodies for flow cytometry and Immunofluorescence; Fig. S1. a Gating strategy for the flow cytometry assay. b Neutrophil-to-monocyte ratio between the ipsilateral and contralateral groups in the bone marrow of male mice. The data are presented as the means ± SD (paired t test, *P < 0.05, n=6-7). c Comparison of neutrophils between ipsilateral and contralateral groups in the bone marrow of different tissues of female mice. The data are presented as the means ± SD (paired t test, **P < 0.01, n=6). d Comparison of monocytes between ipsilateral and contralateral groups in the bone marrow of different tissues of female mice. The data are presented as the means ± SD (paired t test, **P < 0.01, n=6). e Neutrophil-to-monocyte ratio between ipsilateral and contralateral skull marrow of female mice. The data are presented as the means ± SD. (paired t test, **P < 0.01, n=6); Blot images and X-ray image of array. [file 12974_2023_2980_MOESM1_ESM.docx]

**Supplementary Table**

Table S1. Detailed animal characteristics.


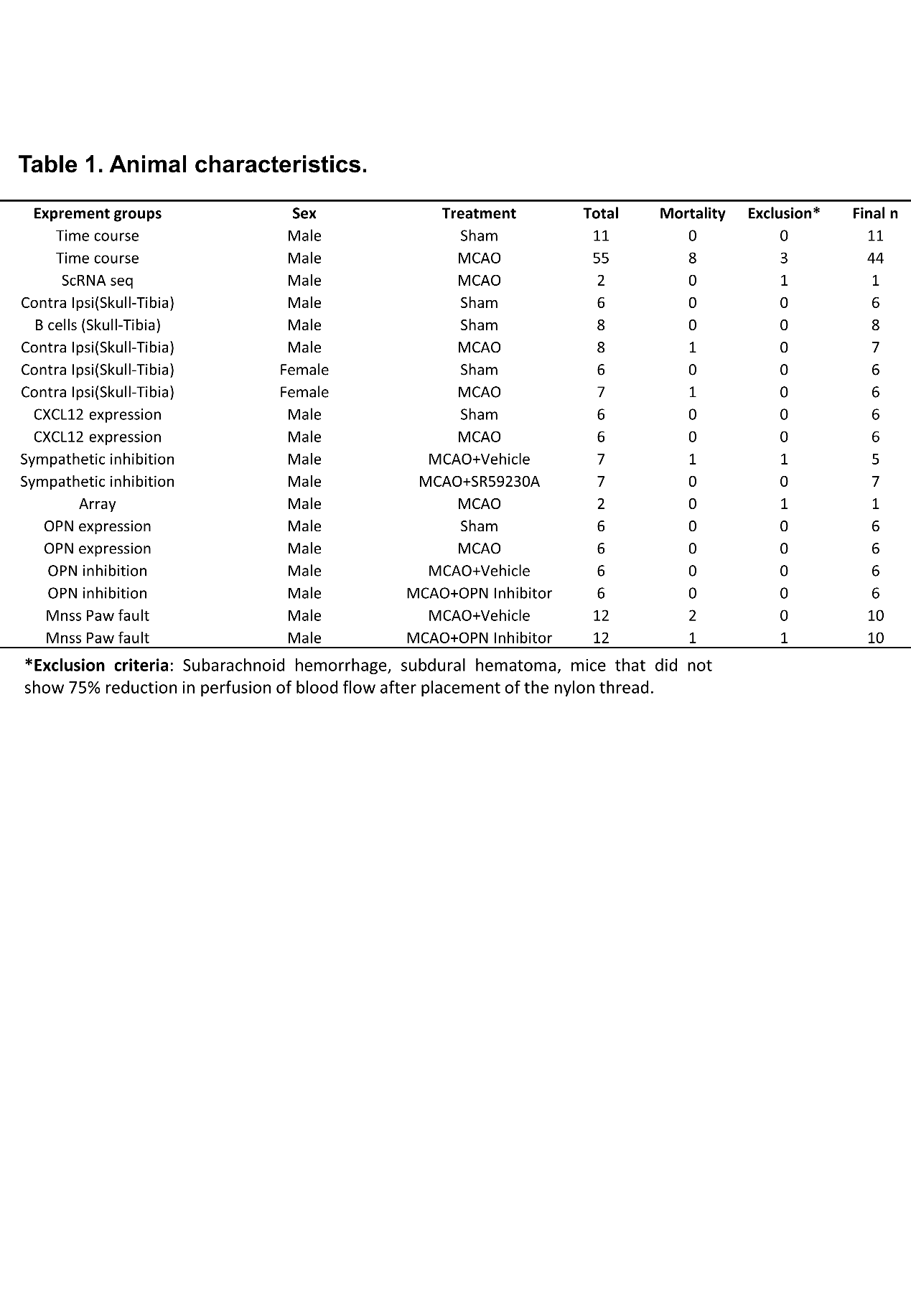


Table S2. List of antibodies for flow cytometry and Immunofluorescence.

| **Target** | **Isotype** | **Conjugate** | **Clone** | **Company** |
| --- | --- | --- | --- | --- |
| CD11b | Rat / IgG2b, kappa | PE-Cyanine7 | M1/70 | Invitrogen |
| CD45 | Mouse / IgG2a, kappa | FITC | 104 | Invitrogen |
| CD115 | Rat / IgG2a, kappa | PE | AFS98 | Invitrogen |
| Ly6c | Rat IgG2c, κ | Brilliant Violet 421 | HK1.4 | BioLegend |
| Ly6g | Rat / IgG2a, kappa | Super Bright 600 | 1A8-Ly6g | Invitrogen |
| c-Kit | Rat IgG2a, κ | Brilliant Violet 785 | 2B8 | BioLegend |
| Sca-1 | Rat IgG2a, κ | Brilliant Violet 605 | D7 | BioLegend |
| Lineage Cocktail | Rat IgG2a/Rat IgG2b | Brilliant Violet 421 | 17A2;RB6-8C5;RA3-6B2; Ter-119; M1/70; | BioLegend |
| B220 | Rat IgG2a, κ | Alexa Fluor 700 | RA3-6B2 | BioLegend |
| Ki67 | Rat / IgG2a, kappa | FITC | SolA15 | Invitrogen |

**Supplementary Figure**

**Supplementary Fig. 1**

**
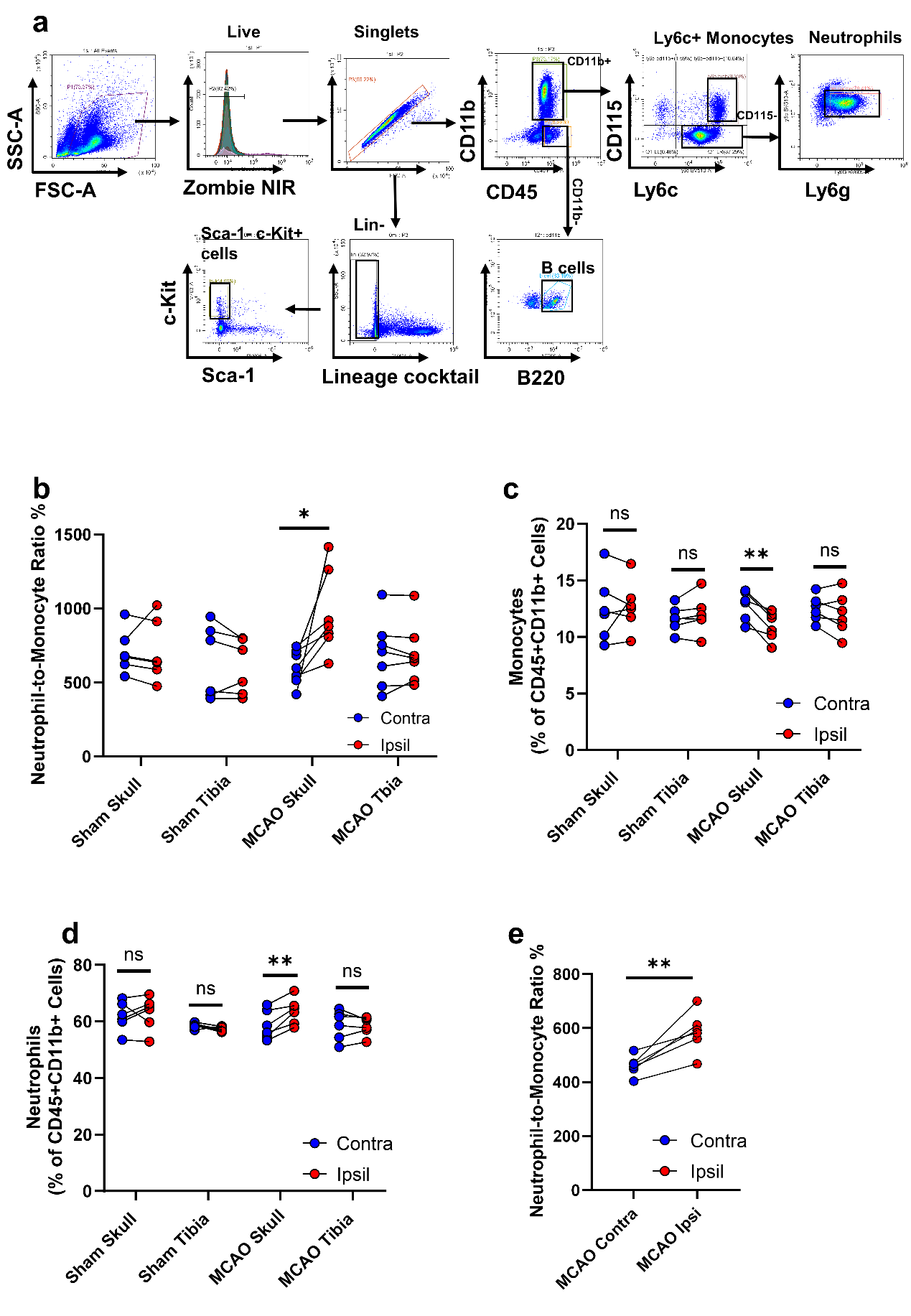
**

**Supplementary Fig. 1**

a) Gating strategy for the flow cytometry assay. b) Neutrophil-to-monocyte ratio between the ipsilateral and contralateral groups in the bone marrow of male mice. The data are presented as the means ± SD (paired t test, *P < 0.05, n=6-7). c) Comparison of neutrophils between ipsilateral and contralateral groups in the bone marrow of different tissues of female mice. The data are presented as the means ± SD (paired t test, **P < 0.01, n=6). d) Comparison of monocytes between ipsilateral and contralateral groups in the bone marrow of different tissues of female mice. The data are presented as the means ± SD (paired t test, **P < 0.01, n=6). e) Neutrophil-to-monocyte ratio between ipsilateral and contralateral skull marrow of female mice. The data are presented as the means ± SD. (paired t test, **P < 0.01, n=6).

**Blot images and X-ray image of array**

**
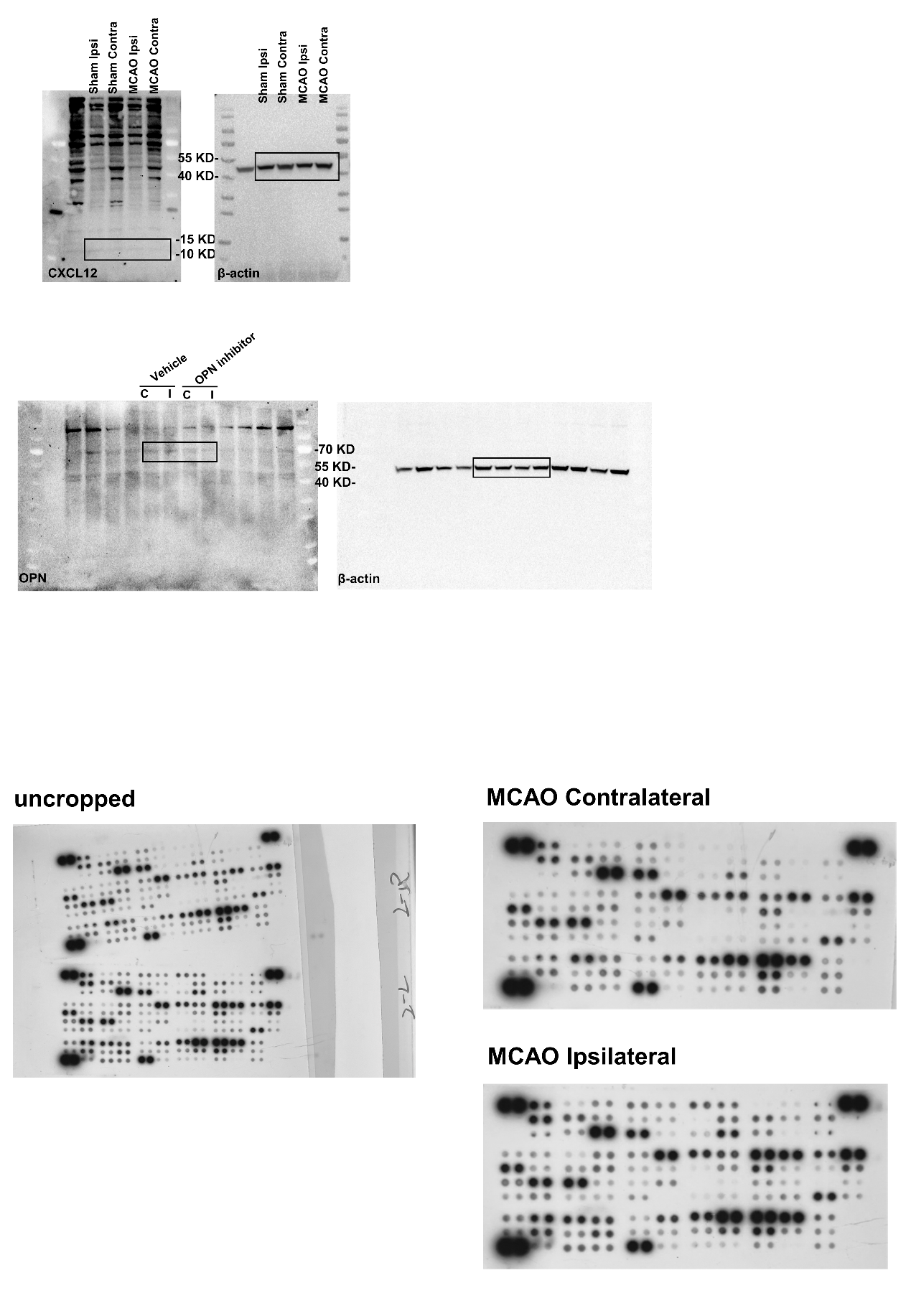
**
